# The 1,4-benzodiazepine Ro5-4864 (4-chlorodiazepam) suppresses multiple pro-inflammatory mast cell effector functions

**DOI:** 10.1186/1478-811X-11-13

**Published:** 2013-02-20

**Authors:** Omid Sascha Yousefi, Thomas Wilhelm, Karin Maschke-Neuß, Marcel Kuhny, Christian Martin, Gerhard J Molderings, Felix Kratz, Bernd Hildenbrand, Michael Huber

**Affiliations:** 1 Medical Faculty, Institute of Biochemistry and Molecular Immunology, RWTH Aachen University, Pauwelsstr. 30, 52074, Aachen, Germany; 2 Medical Faculty, Department of Pharmacology and Toxicology, RWTH Aachen University, Wendlingweg 2, 52074, Aachen, Germany; 3 Institute of Human Genetics, University Hospital of Bonn, Sigmund-Freud-Str. 25, 53127, Bonn, Germany; 4 Division of Macromolecular Prodrugs, Tumor Biology Center, Breisacher Str. 117, 79106, Freiburg, Germany; 5 Department of Clinical Research, Tumor Biology Center, Breisacher Str. 117, 79106, Freiburg, Germany

**Keywords:** Mast cell, Benzodiazepines, Lyn, SHIP1, Mastocytosis, Inflammation, Allergy

## Abstract

Activation of mast cells (MCs) can be achieved by the high-affinity receptor for IgE (FcεRI) as well as by additional receptors such as the lipopolysaccharide (LPS) receptor and the receptor tyrosine kinase Kit (stem cell factor [SCF] receptor). Thus, pharmacological interventions which stabilize MCs in response to different receptors would be preferable in diseases with pathological systemic MC activation such as systemic mastocytosis. 1,4-Benzodiazepines (BDZs) have been reported to suppress MC effector functions. In the present study, our aim was to analyze molecularly the effects of BDZs on MC activation by comparison of the effects of the two BDZs Ro5-4864 and clonazepam, which markedly differ in their affinities for the archetypical BDZ recognition sites, i.e., the GABA_A_ receptor and TSPO (previously termed peripheral-type BDZ receptor). Ro5-4864 is a selective agonist at TSPO, whereas clonazepam is a selective agonist at the GABA_A_ receptor. Ro5-4864 suppressed pro-inflammatory MC effector functions in response to antigen (Ag) (degranulation/cytokine production) and LPS and SCF (cytokine production), whereas clonazepam was inactive. Signaling pathway analyses revealed inhibitory effects of Ro5-4864 on Ag-triggered production of reactive oxygen species, calcium mobilization and activation of different downstream kinases. The initial activation of Src family kinases was attenuated by Ro5-4864 offering a molecular explanation for the observed impacts on various downstream signaling elements. In conclusion, BDZs structurally related to Ro5-4864 might serve as multifunctional MC stabilizers without the sedative effect of GABA_A_ receptor-interacting BDZs.

## Background

1,4-Benzodiazepines (BDZs) are clinically used as anxiolytic, hypnotic, anti-convulsive, and muscle relaxing drugs [1-4]. BDZs are lipophilic and readily cross cell membranes. There are two major types of BDZ recognition sites. The first site is part of the GABA_A_ receptor complex found in cells of the central nervous system [5] and, hence, is termed central-type BDZ receptor. The other one is an ubiquitously expressed transmembrane protein of the outer mitochondrial membrane (OMM) termed translocator protein (18 kDa) (TSPO) [6] (previously named peripheral-type BDZ receptor [7]). Interaction studies revealed that TSPO is associated with the OMM protein voltage-dependent anion channel (VDAC) and the inner mitochondrial membrane (IMM) protein adenine nucleotide transporter (ANT) [8] and the requirement for both TSPO and VDAC for BDZ binding has been suggested. Most BDZs clinically used possess nanomolar affinity for the central-type receptor, but only milli- to micromolar affinities for TSPO. However, there are also BDZs available with high affinity and selectivity for TSPO (e.g. Ro5-4864 = 4-chlorodiazepam) [9], thus, allowing the analysis of the potential involvement of TSPO function in biological processes.

Expression of TSPO has also been described in mast cells (MCs) [10-12]. MCs are hematopoietic, tissue-resident cells, which are involved in various physiological as well as pathophysiological scenarios. They are very important players in innate and adaptive immune responses, inflammation, and tissue changes [13,14]. Important reactions during these processes are allergen-triggered degranulation of preformed mediators (e.g. histamine and proteases) and lipopolysaccharide (LPS)-induced production of pro-inflammatory cytokines (e.g. IL-6 and TNF-α), respectively. In addition to the allergy-relevant high-affinity receptor for IgE (FcεRI) and the LPS receptor (TLR4), the receptor tyrosine kinase Kit represents another important signaling system, which regulates MC differentiation, proliferation, survival, chemotaxis, and production of pro-inflammatory cytokines [15].

BDZs have been reported to inhibit MC effector functions: Midazolam suppressed substance P-induced chemotaxis as well as degranulation [16] of canine MCs. Diazepam and midazolam inhibited proliferation of murine MCs as well as pro-inflammatory mediator release from these cells [17]. With respect to systemic MC activation disease (MCAD) [17,18], the clinical efficacy of the BDZs flunitrazepam, diazepam, bromazepam, and midazolam for the treatment of MC mediator-induced symptoms has been reported [18,19]. The TSPO-selective BDZ Ro5-4864 was shown to inhibit concanavalin A-induced serotonin release from as well as ^45^Ca uptake into rat MCs, whereas the GABA_A_-receptor-selective BDZ clonazepam only had a slight impact on serotonin release and did not affect ^45^Ca uptake [11]. In addition, diazepam, Ro5-4864, and flunitrazepam were demonstrated to reduce NECA-induced IL-8 production in human MC leukemia cells [20]. Since on the one hand MCs did not express GABA_A_ receptors in previous investigations and on the other hand most BDZs, such as flunitrazepam, diazepam, and midazolam possess considerable affinity for TSPO [7,9,12], it was conceivable that the inhibitory effects of BDZs on MCs may be due to their binding to TSPO in MCs. In this context the aim of the present study was to analyze the molecular processes underlying the inhibitory action of BDZs in MCs by using two selective BDZs: Ro5-4864 (4-chlorodiazepam) that possesses high affinity for TSPO but has only low affinity for GABA_A_ receptors and clonazepam, a high-affinity ligand for GABA_A_ receptors with only low affinity for TSPO [12].

We show here that Ro5-4864 but not clonazepam inhibited antigen (Ag)-triggered degranulation as well as Ag-, LPS- or SF-induced pro-inflammatory cytokine production. In addition, Ro5-4864 inhibited allergen-induced bronchoconstriction in precision-cut lung slices. Moreover, Ag-triggered Ca^2+^ mobilization and production of reactive oxygen species (ROS) were suppressed by Ro5-4864. By expressing a fluorescent TSPO fusion protein and using confocal microscopy, we were not able to detect a plasmalemnal localization of the TSPO-containing fusion protein in MCs which has been observed previously in some other cell types [21,22]. Analysis of early Ag-triggered signaling events suggested Ro5-4864-dependent attenuation of Src family kinases (SFKs), which represent very early signaling molecules active in the chain of FcεRI signaling. Hence, attenuation of SFKs by direct inhibition and/or indirectly by targeting a so far unidentified upstream plasmalemnal recognition site could be the reason for the observed suppression of pro-inflammatory MC responses.

## Results

### Ro5-4864 inhibits mast cell degranulation

**Figure 1:**
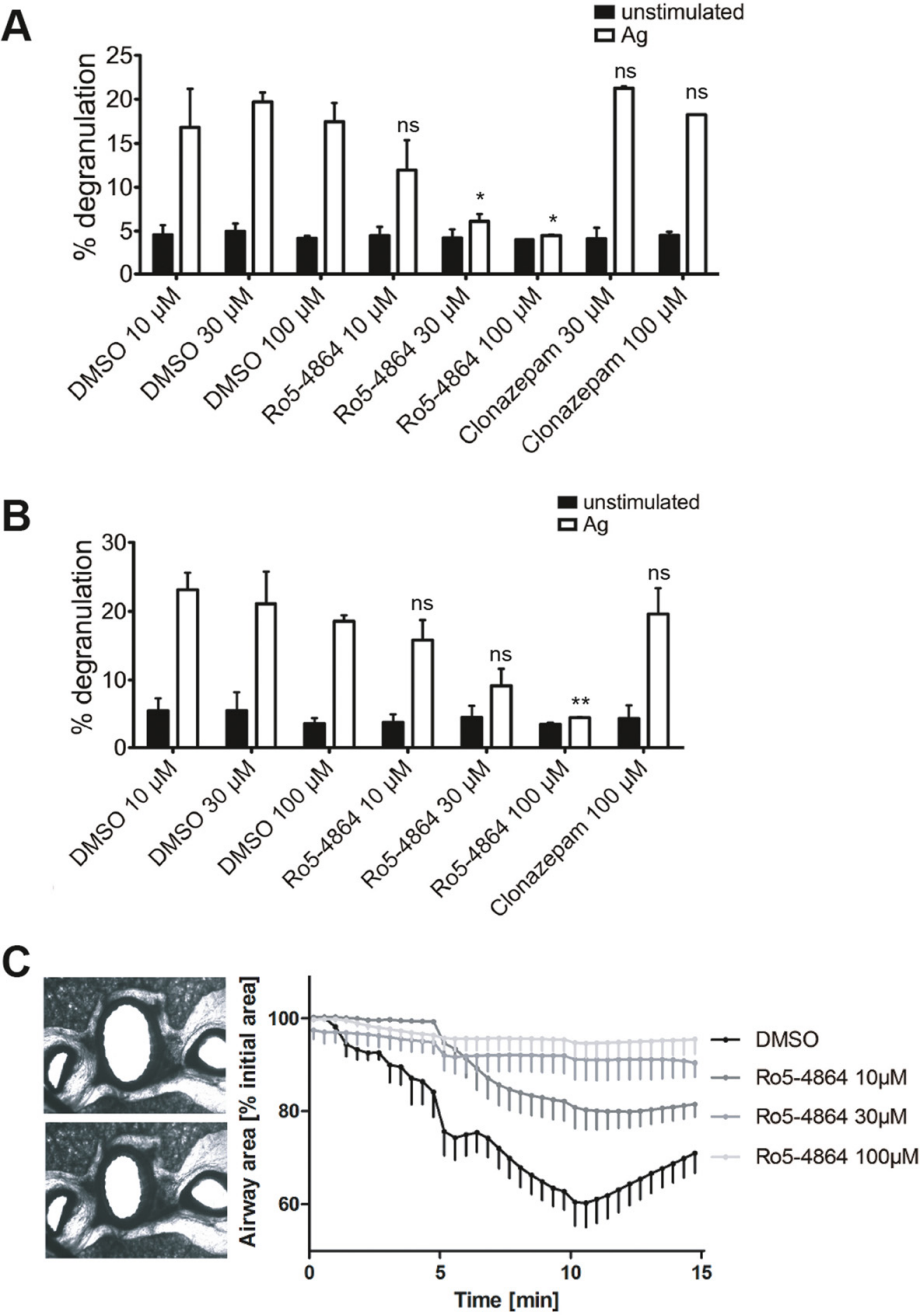
**Ro5-4864 inhibits MC degranulation. (A)** IgE-loaded BMMCs were pretreated for 20 min with the indicated concentrations of BDZs (Ro5-4864 and clonazepam) or a corresponding amount of DMSO and then either stimulated (white bars) with 20 ng/ml Ag (DNP-HSA) or left unstimulated (black bars) for another 20 min. Degranulation was measured by β-hexosaminidase assay. Each bar is the mean of duplicates ± SEM. Comparable results were obtained with cells from different cultures (n≥3). **(B)** IgE-loaded PMCs were treated as in (**A**) and degranulation was measured. Each bar is the mean of duplicates ± SEM. Marks of significance (“asterisks”) relate to the respective vehicle (DMSO) control. Each vehicle control contains the volume of DMSO necessary to dissolve the drugs in the respective BDZ samples. **(C)** Airway contractions to ovalbumin in rat precision-cut lung slices. On the left upper image airway under control condition is shown, whereas in the left lower image the airway has contracted to ovalbumin (10 μg/ml). On the right side kinetics of airway contractions to a single dose of OVA (10 μg/ml, n=5) in the presence and absence of Ro5-4864 (10 μM, 30 μM and 100 μM) is shown. Contractions are expressed as the decrease of airway area (%) compared to the initial airway area. Data (n=5) are presented as mean ± SEM. The area under the curve was compared by a one-way ANOVA followed by a Dunnett-test for multiple comparisons. All Ro5-4864 concentrations showed a statistical significance compared to control.

IgE-loaded BMMCs were stimulated with Ag (DNP-HSA) in the presence of vehicle (DMSO), Ro5-4864 or clonazepam and degranulation was measured by means of β-hexosaminidase assays. As shown in Figure 1A, Ro5-4864 in a concentration-dependent manner inhibited Ag-triggered degranulation, whereas clonazepam did not show an effect different from the vehicle control. To verify these data in a further MC model, PMCs were treated and stimulated in a comparable fashion and degranulation was measured. PMCs are an accepted model for serosal MCs [23]. Again, clonazepam failed to decrease Ag-triggered degranulation, whereas Ro5-4864 inhibited degranulation in a concentration-dependent manner (Figure 1B). The activity of IgE-loaded BMMCs and PMCs in the absence of Ag as determined by spontaneous degranulation was not influenced by the vehicle (DMSO), Ro5-4864 or clonazepam (Figure 1A and B). Next, the effect of Ro5-4864 on allergen-induced bronchoconstriction in rat lung slices was investigated. Lung slices have an intact microanatomy and represent whole lung function in a reproducible way [24]. In precision-cut lung slices, which were pretreated with serum from sensitized animals, allergen-induced bronchoconstriction was inhibited by Ro5-4864 in a concentration-dependent manner (Figure 1C). These data suggest that Ag-triggered MC activation can be suppressed by Ro5-4864.

### Ro5-4864 suppresses Ag-triggered Ca^2+^ influx as well as ROS production

**Figure 2:**
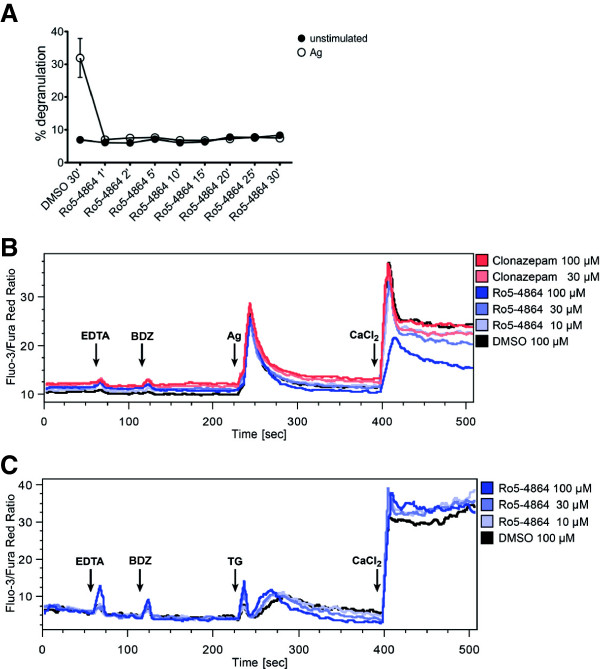
**Ro5-4864 suppresses Ag-triggered Ca^2+^ flux. (A)** BMMCs were pretreated for the indicated times with 100 μM Ro5-4864 or for 30 min with a corresponding amount of DMSO and then either stimulated with Ag (DNP-HSA) (open symbols) or left unstimulated (solid symbols) for another 10 min. Degranulation was measured by β-hexosaminidase assay. Each value is the mean of duplicates ± SEM. **(B)** Intracellular Ca^2+^ was measured in BMMCs by flow cytometry using the Ca^2+^-sensitive fluorescent dyes fluo-3 and fura red. Steady-state fluorescence was determined for 1 min before 1 mM EDTA (first arrow) was added for 1 min to chelate extracellular Ca^2+^. Denoted BZDs or DMSO were added (second arrow) and incubated for 2 min. Cells were then stimulated with 200 ng/ml Ag (third arrow) and the resulting Ca^2+^ response derived from intracellular store depletion was measured for 3 min. Finally, 2 mM CaCl_2_ was added (fourth arrow) to replenish extracellular Ca^2+^ stores and the resulting SOC influx was measured for 2 min. Comparable results were obtained with cells from different cultures (n≥3). **(C)** Measurements were performed as described under (**B**) with the exception that cells were not stimulated with Ag, but with the SERCA inhibitor thapsigargin (TG). Comparable results were obtained with cells from two different cultures.

**Figure 3:**
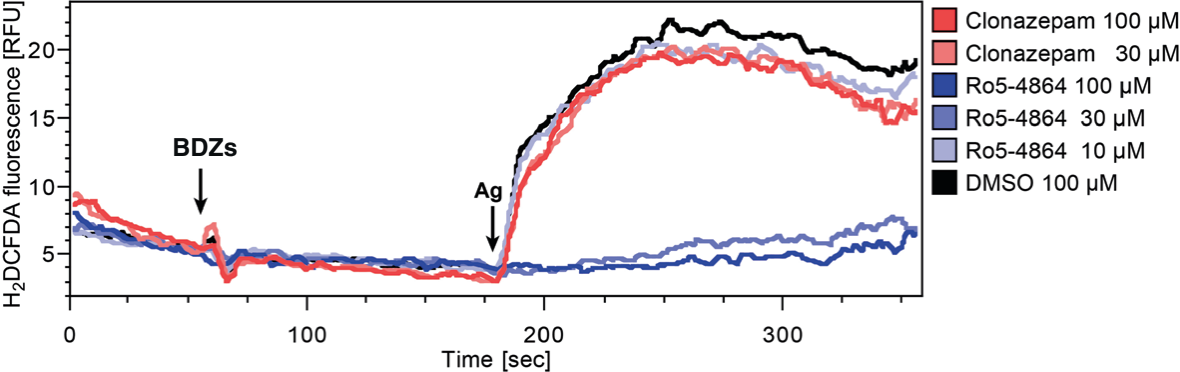
**Ro5-4864 inhibits Ag-triggered ROS production.** Intracellular ROS were measured in BMMCs by flow cytometry using the ROS-sensitive fluorescent dye H_2_DCFDA. Background fluorescence was determined for 1 min before indicated BDZs or DMSO were added (first arrow) and incubated for 2 min. Cells were then stimulated with 200 ng/ml Ag (second arrow) and the resulting intracellular ROS response was measured for 3 min. Comparable results were obtained with cells from two different cultures.

It is well known that Ca^2+^ influx is mandatory for a degranulation reaction to occur [25]. Furthermore, production of reactive oxygen species (ROS) has been demonstrated to be important for Ag-triggered degranulation [26,27]. The following Ca^2+^flux and ROS measurements were carried out by means of flow cytometry. Due to the respective processing of the cells it was desirable to keep the pretreatment time with BDZs as short as possible. Thus, the shortest effective incubation time with Ro5-4864 to suppress MC degranulation was determined. As shown in Figure 2A, a pretreatment time even as short as 1 min resulted in complete inhibition of Ag-triggered MC degranulation by Ro5-4864. Consequently, BDZ treatment for Ca^2+^ flux and ROS measurements was reduced to 2 min. Ag-triggered Ca^2+^ mobilization is a two step process comprising initial release of intracellular Ca^2+^ from the endoplasmic reticulum (ER) via inositol-1,4,5-trisphosphate-gated Ca^2+^ channels and subsequent influx of extracellular Ca^2+^ via store-operated Ca^2+^ (SOC) channels [28]. BMMCs were pretreated with BDZs and stimulated with Ag in the presence of the Ca^2+^ chelator EDTA to allow measurement of release of intracellular Ca^2+^ only. As soon as the intracellular Ca^2+^ concentration had returned to background levels, CaCl_2_ was added to the cells to measure Ca^2+^ influx through opened SOC channels. Neither Ro5-4864 nor clonazepam significantly influenced intracellular Ca^2+^ release (Figure 2B). However, Ro5-4864 concentration-dependently attenuated the influx of extracellular Ca^2+^ (Figure 2B). Since in particular extracellular Ca^2+^ influx is mandatory for MC degranulation [26] these results correlate with Ro5-4864′s effect on degranulation. SOC influx can also be triggered by thapsigargin, an inhibitor of the sarcoplasmic-endoplasmic reticulum Ca^2+^ ATPase (SERCA), which induces receptor-independent, passive release of Ca^2+^ from the ER and thus triggers SOC influx as well as degranulation [29]. Intriguingly, Ro5-4864 did not suppress thapsigargin-induced Ca^2+^ influx, but even slightly enhanced it (Figure 2C), suggesting that Ro5-4864 specifically influences an FcεRI-triggered signaling mechanism important for Ca^2+^ mobilization. It has been shown previously that Ag-triggered SOC entry and degranulation are dependent on intracellular ROS production [27]. In agreement with our data on Ro5-4864 action, pretreatment with Ro5-4864 inhibited Ag-induced ROS production in a concentration-dependent manner (Figure 3). In contrast, clonazepam did not influence Ag-triggered ROS production (Figure 3).

### Ro5-4864 suppresses pro-inflammatory cytokine production in mast cells

**Figure 4:**
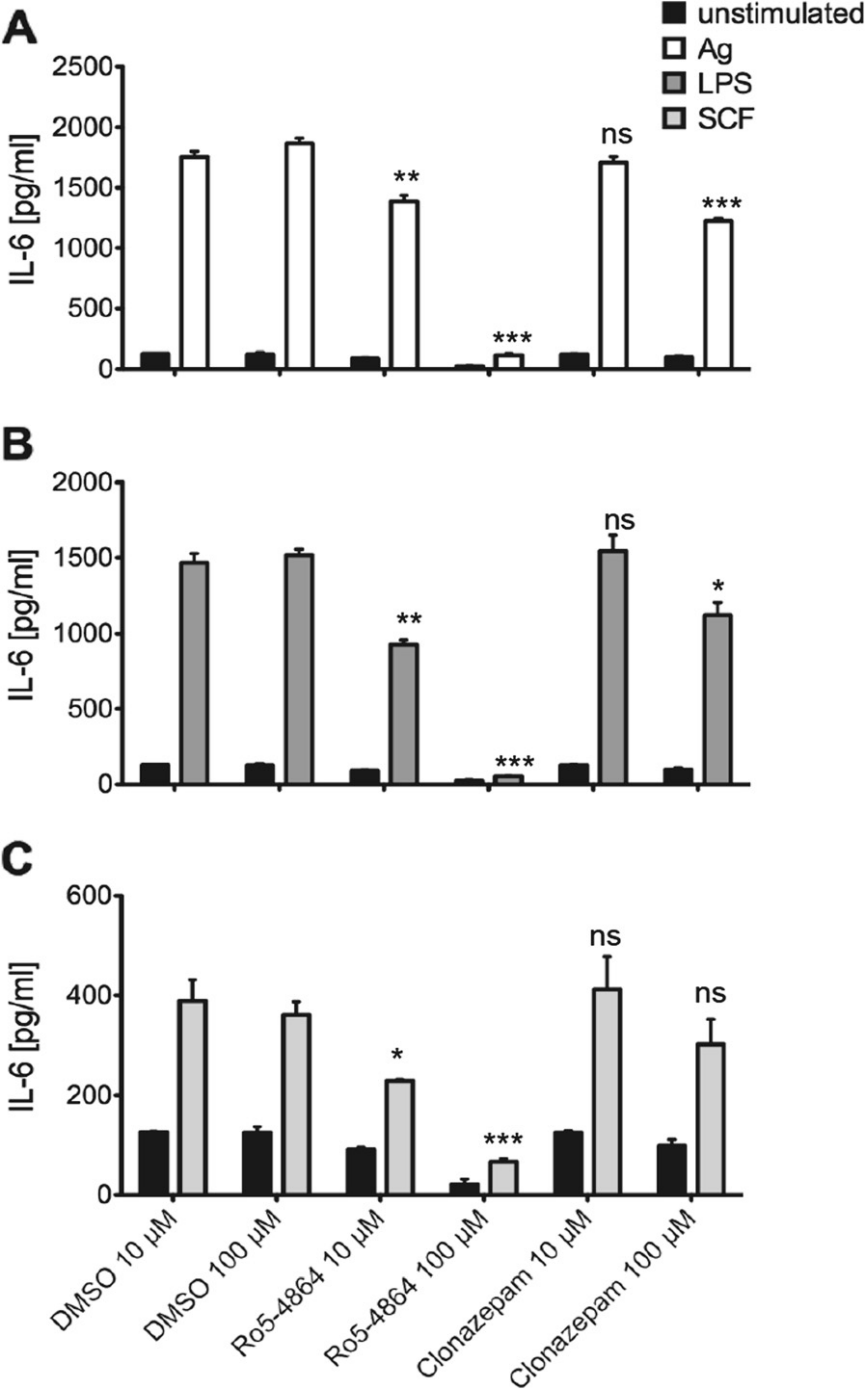
**Suppression of Ag-, LPS-, and SCF-triggered IL-6 production by Ro5-4864.** IgE-loaded BMMCs were pretreated with DMSO, Ro5-4864 or clonazepam for 20 min and subsequently stimulated with 20 ng/ml Ag **(A)**, 5 μg/ml LPS **(B)** or 150 ng/ml SCF **(C)** for 3 h or left unstimulated. Subsequently, IL-6 concentrations in the supernatants were determined by ELISA. Each bar is the mean of triplicates ± SEM. Comparable results were obtained with cells from different cultures (n≥3). Marks of significance (“asterisks”) relate to the respective vehicle (DMSO) control.

**Figure 5:**
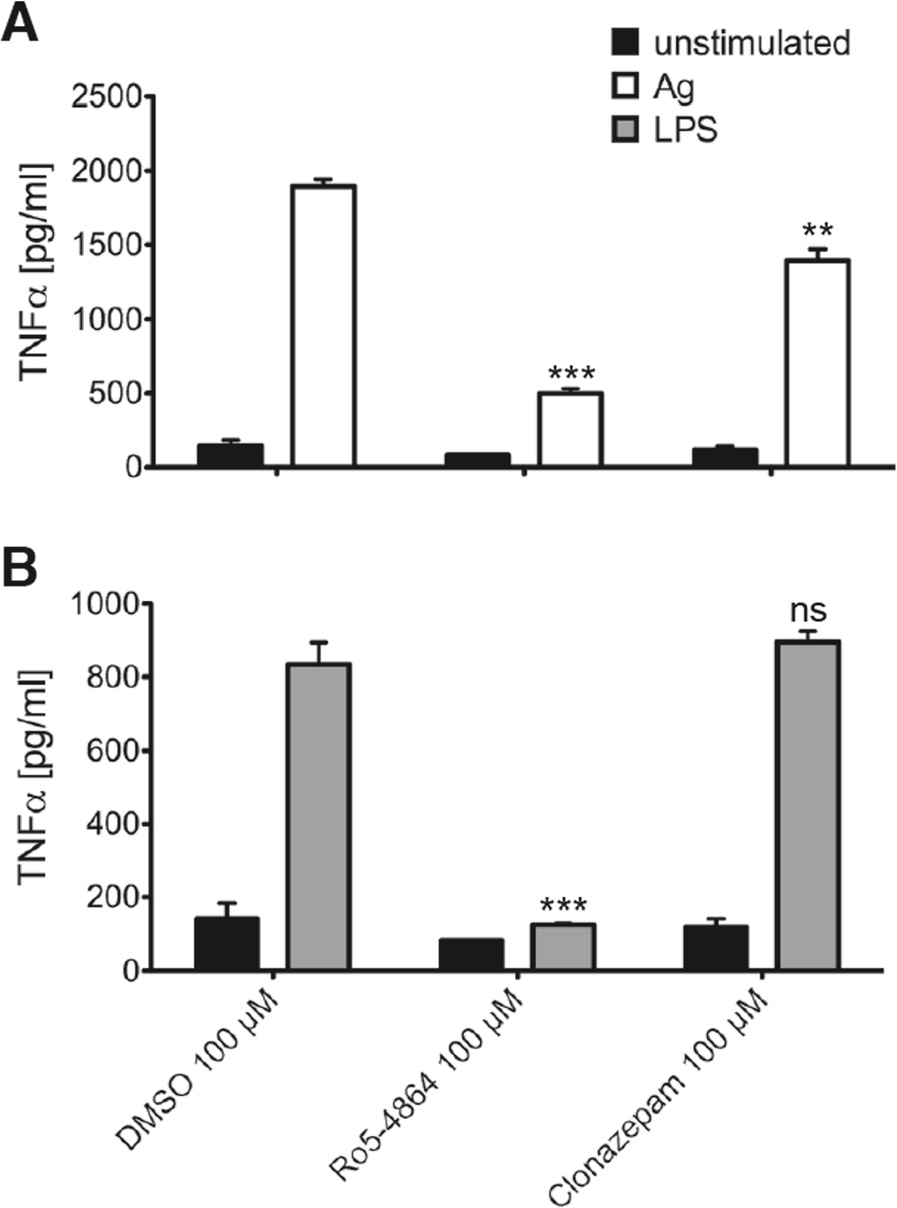
**Ro5-4864 inhibits TNF-α production in response to Ag and LPS.** IgE-loaded BMMCs were pretreated with DMSO, Ro5-4864 or clonazepam for 20 min and subsequently stimulated with 20 ng/ml Ag **(A)** or 5 μg/ml LPS **(B)** for 3 h or left unstimulated. TNF-α concentrations in the supernatants were determined by ELISA. Each bar is the mean of triplicates ± SEM. Comparable results were obtained with cells from different cultures (n≥3). Marks of significance (“stars”) relate to the respective vehicle (DMSO) control.

Degranulation represents an immediate early response to Ag stimulation taking place within the first minutes. Another important pro-inflammatory response is the production of cytokines (e.g. IL-6 and TNF-α), which is a kinetically later event; significant amounts of produced and secreted cytokines are measurable within 1.5 – 3 h after Ag treatment. Moreover, pro-inflammatory cytokines can also be produced in response to LPS and SCF, whereas degranulation is a response particularly triggered by Ag. Here, we sought to analyze the effects of Ro5-4864 and clonazepam on pro-inflammatory cytokine production after stimulation of BMMCs with different ligands. As shown in Figure 4, Ro5-4864 inhibited production of IL-6 in a concentration-dependent manner in BMMCs stimulated with Ag (Figure 4A), LPS (Figure 4B), and SCF (Figure 4C). Although a certain suppression of IL-6 production was found also in the presence of the highest concentration (100 μM) of clonazepam, there was a significant difference in efficiency between Ro5-4864 and clonazepam (Figure 4). A comparable pattern was observed when investigating the effects of BDZs on production of TNF-α in response to Ag (Figure 5A) and LPS (Figure 5B). Thus, our data show that Ro5-4864, in contrast to clonazepam, is able to partially suppress pro-inflammatory MC activation (degranulation and cytokine production) in response to different ligand/receptor systems.

### Ro5-4864 attenuates activation of the PI3K pathway

**Figure 6:**
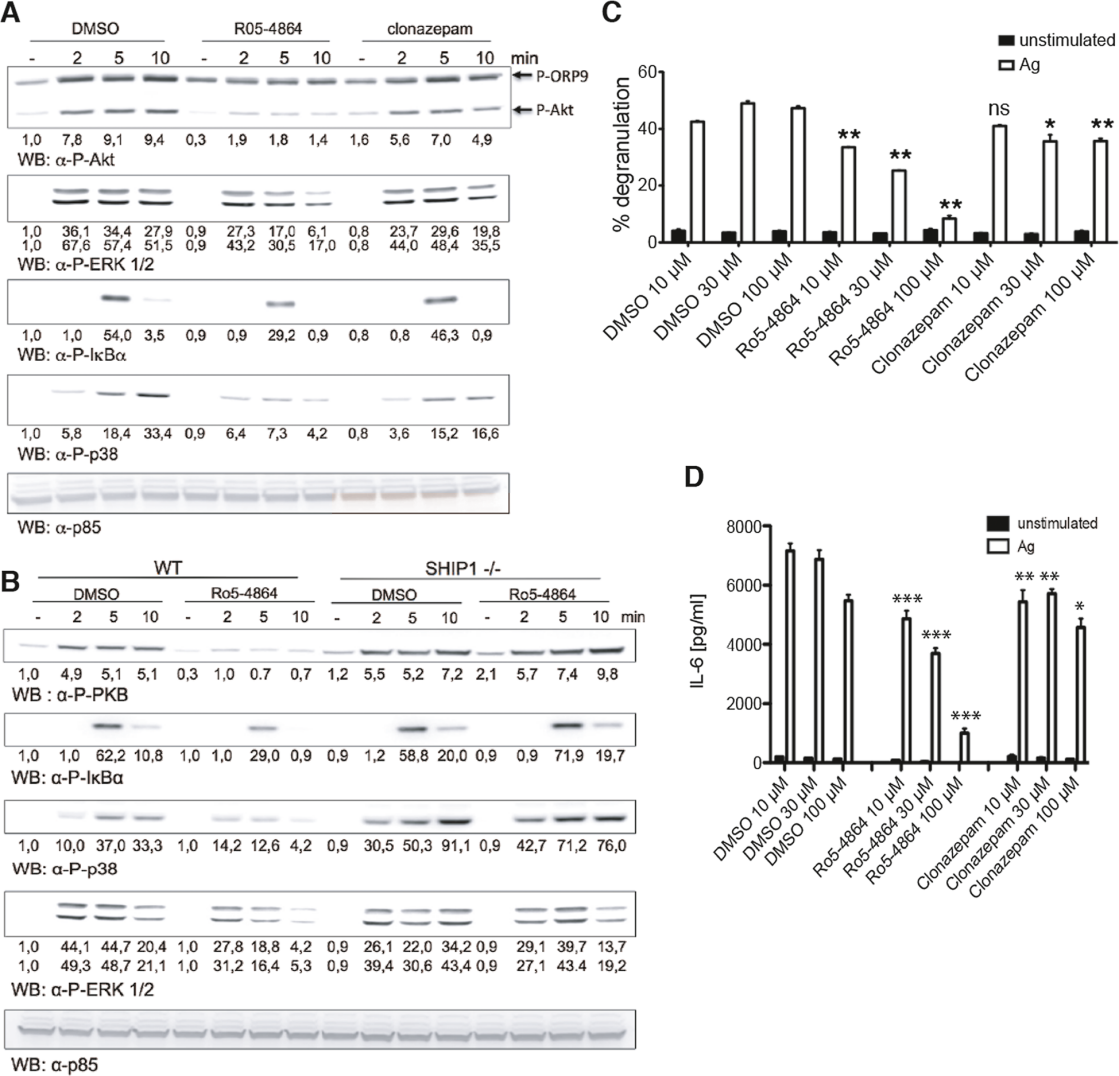
**Ro5-4864 treatment attenuates activation of the PI3K pathway. (A)** IgE-loaded BMMCs were pretreated for 20 min with 100 μM Ro5-4864, 100 μM clonazepam or the respective amount of DMSO and stimulated with Ag (DNP-HSA, 20 ng/ml) for the indicated times or left unstimulated. Subsequently, cellular lysates were analyzed by immunoblotting using antibodies against P-Akt (top panel), P-ERK1/2 (2^nd^ panel from top), P-IκBα (3^rd^ panel from top), P-p38 (4^th^ panel from top), and p85 (bottom panel, loading control). **(B)** IgE-loaded SHIP1+/+ and SHIP1−/− BMMCs were pretreated for 20 min with 100 μM Ro5-4864 or DMSO and stimulated for the indicated times with 20 ng/ml Ag. Cellular lysates were analyzed as described in (**A**). Comparable results were obtained with cells from different BMMC cultures. Densitometry was performed and relative expression levels are indicated under each band. **(C)** IgE-loaded SHIP1-deficient BMMCs were pretreated for 20 min with the indicated concentrations of Ro5-4864, clonazepam or a corresponding amount of DMSO and then either stimulated with 20 ng/ml Ag (DNP-HSA) or left unstimulated for another 20 min. Degranulation was measured by β-hexosaminidase assay. Each bar is the mean of duplicates ± SEM. Comparable results were obtained with cells from different cultures (n≥3). **(D)** IgE-loaded SHIP1-deficient BMMCs were pretreated for 20 min with the indicated concentrations of Ro5-4864, clonazepam or a corresponding amount of DMSO and then stimulated with 20 ng/ml Ag or left unstimulated for 3 h. IL-6 concentrations in the supernatants were determined by ELISA. Each bar is the mean of triplicates ± SEM. Comparable results were obtained with cells from different cultures (n≥3). Marks of significance (“asterisks”) relate to the respective vehicle (DMSO) control.

Subsequently, we were interested in the molecular signaling processes underlying the influence of Ro5-4864 on MC effector functions. It has been shown by others and our laboratory that Ag-induced activation of the PI3K pathway is crucial for degranulation [26,30]. Therefore, we compared the effects of Ro5-4864 and clonazepam on Ag-triggered phosphorylation of Akt at S473, which is a well-known step in PI3K-dependent signal transduction. As shown in Figure 6A, correlating with the effects on degranulation, Ro5-4864 pretreatment did result in markedly reduced Akt phosphorylation, whereas such an effect was not observed with clonazepam. A second protein of approximately 80 kDa can be detected in MCs by the anti-P-Akt (S473) antibody. This represents ORP9 phosphorylated by PKC-β at S287 [31]. The observed phosphorylation pattern suggests that PKC-β activity is suppressed by Ro5-4864 treatment as well, whereas clonazepam has minimal activity (Figure 6A). Interestingly, activation of the MEK-Erk pathway (measured by detecting bis-phosphorylation of Erk1/2 at T202/Y204) appeared to be less influenced by Ro5-4864 treatment (Figure 6A), suggesting that Ro5-4864 does not suppress MC activation in a generalized manner. Since phosphorylation of Akt is dependent on PI3K-mediated production of the second messenger PIP_3_, the observed effect of Ro5-4864 treatment could in part be due to attenuation of PI3K activation or to enhanced activation of the PIP_3_ phosphatase SHIP1, the prominent counter-player in MCs within the PI3K pathway [32]. To address this, wild-type and SHIP1-deficient BMMCs were stimulated with Ag in the presence of vehicle or Ro5-4864 and S473 phosphorylation of Akt was analyzed by Western blotting. In contrast to wild-type cells, Ro5-4864 pretreatment did not affect Akt S473 phosphorylation in SHIP1-deficient BMMCs, suggesting that the effect of Ro5-4864 was due to affecting SHIP1 activation rather than PI3K activation (Figure 6B). These data suggested that Ro5-4864 should not be able to suppress Ag-triggered degranulation in SHIP1-deficient BMMCs. Thus, SHIP1-deficient BMMCs were pretreated with vehicle or Ro5-4864 and degranulation in response to Ag was measured. Unexpectedly, the inhibitory effect of Ro5-4864 was also observed in SHIP1-deficient BMMCs (Figure 6C).

Interestingly, a comparable concentration-dependent pattern was observed when analyzing the effect of Ro5-4864 on Ag- or LPS-triggered IL-6 production in SHIP1-deficient BMMCs (Figure 6D and Additional file 1: Figure S1). Pro-inflammatory cytokine production downstream of the FcεRI strongly depends on the NFκB as well as the p38 MAPK pathways [33]. Whereas Ro5-4864 treatment resulted in suppressed activation of p38 (measured by bis-phosphorylation at T180/Y182) in response to Ag in wild-type cells, no such effect was observed in SHIP1-deficient BMMCs (Figure 6B). In addition, phosphorylation of IκBα at S32 indicating activation of the NFκB pathway was slightly attenuated by Ro5-4864 treatment in wild-type BMMCs, which was not the case in SHIP1-deficient cells (Figure 6B). These data suggest that Ro5-4864 suppresses MC activation and effector functions independently of its effect on the PI3K, p38, and/or NFκB pathways.

### Ro5-4864 interferes with the tyrosine phosphorylation response in mast cells

**Figure 7:**
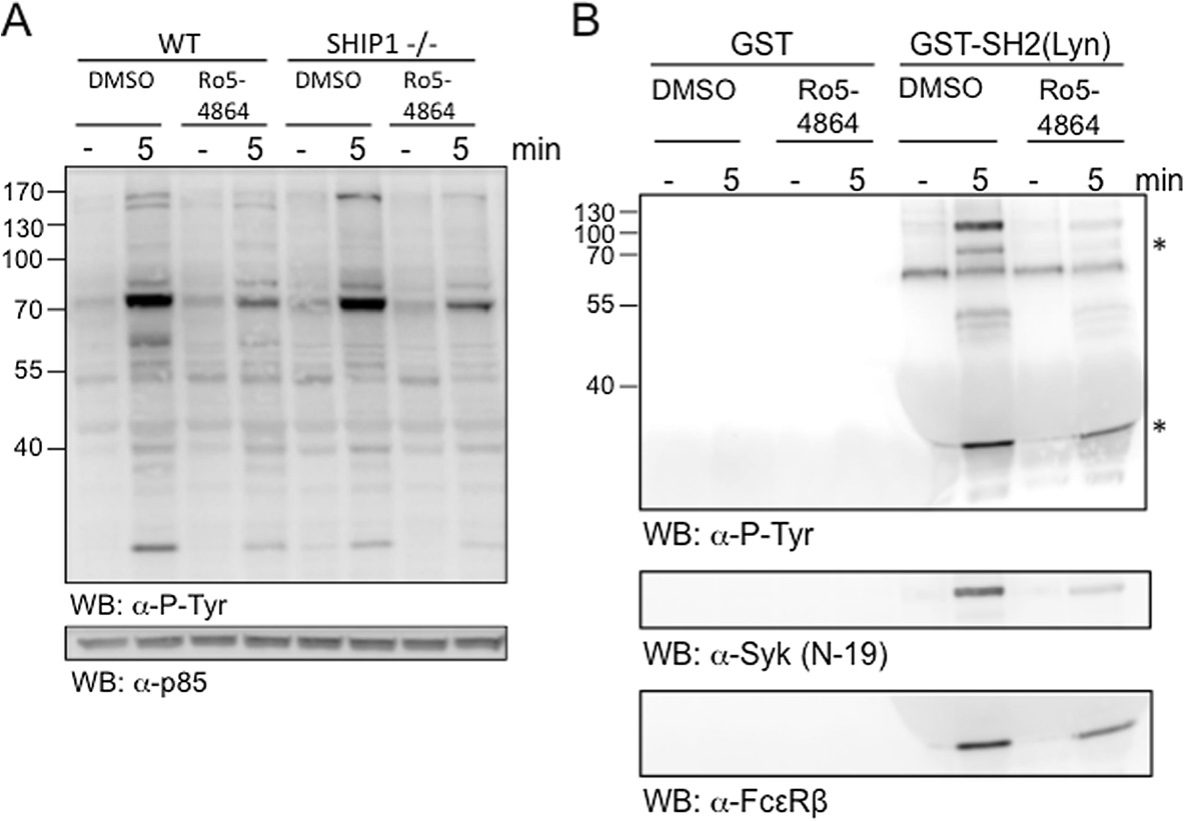
**Ro5-4864 affects Ag-triggered tyrosine phosphorylation events in wild-type and SHIP1-deficient BMMCs. (A)** IgE-loaded wild-type and SHIP1-deficient BMMCs were pretreated for 20 min with 100 μM Ro5-4864 or the respective amount of DMSO and stimulated with Ag (DNP-HSA, 20 ng/ml) for 5 min or left unstimulated. Subsequently, cellular lysates were analyzed by immunoblotting using antibodies against phosphotyrosine (upper panel) and p85 (lower panel, loading control). **(B)** Wild-type BMMCs were treated as under (**A**) and cellular lysates were subjected to pull-down reactions with GST or GST-SH2(Lyn) proteins. Precipitated proteins were analyzed by immunoblotting against phosphotyrosine (top panel), Syk (middle panel), and FcεRIβ-chain (bottom panel). Usage of equal amounts of GST proteins was verified by Coomassie staining (data not shown). Comparable results were obtained with cells from different BMMC cultures.

The comparative signaling data obtained in wild-type and SHIP1-deficient BMMCs suggested that Ro5-4864 affects signaling events, which are activated independently of PI3K activation and/or SHIP1 presence. Protein tyrosine phosphorylations represent the earliest signaling events in response to Ag [34]. In this respect, BDZs were found to have the potential to inhibit the tyrosine kinase Src [35]. Moreover, the concentrations of Ro5-4864 needed for the observed inhibitory effects (10–100 μM) appeared too high for a specific pharmacologic effect on TSPO [36]. Since among the first kinases activated in response to Ag are the SFKs Lyn and Fyn, we compared the effect of Ro5-4864 on Ag-induced tyrosine phosphorylation events in wild-type and SHIP1-deficient BMMCs. Indeed, in both wild-type and SHIP1-deficient BMMCs Ro5-4864 pretreatment resulted in attenuation of certain Ag-induced tyrosine phosphorylation events (Figure 7A). Since this measurement is not target-selective, we decided to specifically look at the tyrosine phosphorylation status of the β-chain of the FcεRI, a major Lyn target. A GST-fusion protein containing the SH2-domain of Lyn can be used to pull down tyrosine-phosphorylated FcεRIβ [37]. Thus, BMMCs were stimulated with Ag in the presence or absence of Ro5-4864, respective lysates were subjected to GST-SH2(Lyn) pull-down, and interacting proteins were analyzed by anti-phosphotyrosine immunoblotting. Corroborating the data shown in Figure 7A, significantly less tyrosine-phosphorylated proteins were pulled-down from lysates of Ro5-4864-treated cells (Figure 7B). Amongst these, two proteins of 35 kDa and 70 kDa were detected (asterisks), most likely representing FcεRIβ and the tyrosine kinase Syk, respectively. The latter has also been found to be a Lyn target [38]. Reprobing the membrane with anti-FcεRIβ and anti-Syk antibodies confirmed reduced pull-down of these proteins with GST-SH2(Lyn) from lysates of Ro5-4864-treated cells (Figure 7B), indicating reduced tyrosine phosphorylation of these proteins. The pull-down reactions were not directly influenced by Ro5-4864 (data not shown). These data suggest that Ro5-4864 is able to suppress tyrosine kinase activity, most likely of SFKs, which might result in attenuated Ag-triggered activation of MC effector functions such as degranulation and pro-inflammatory cytokine production.

### TSPO is expressed in mitochondria of mast cells

**Figure 8:**
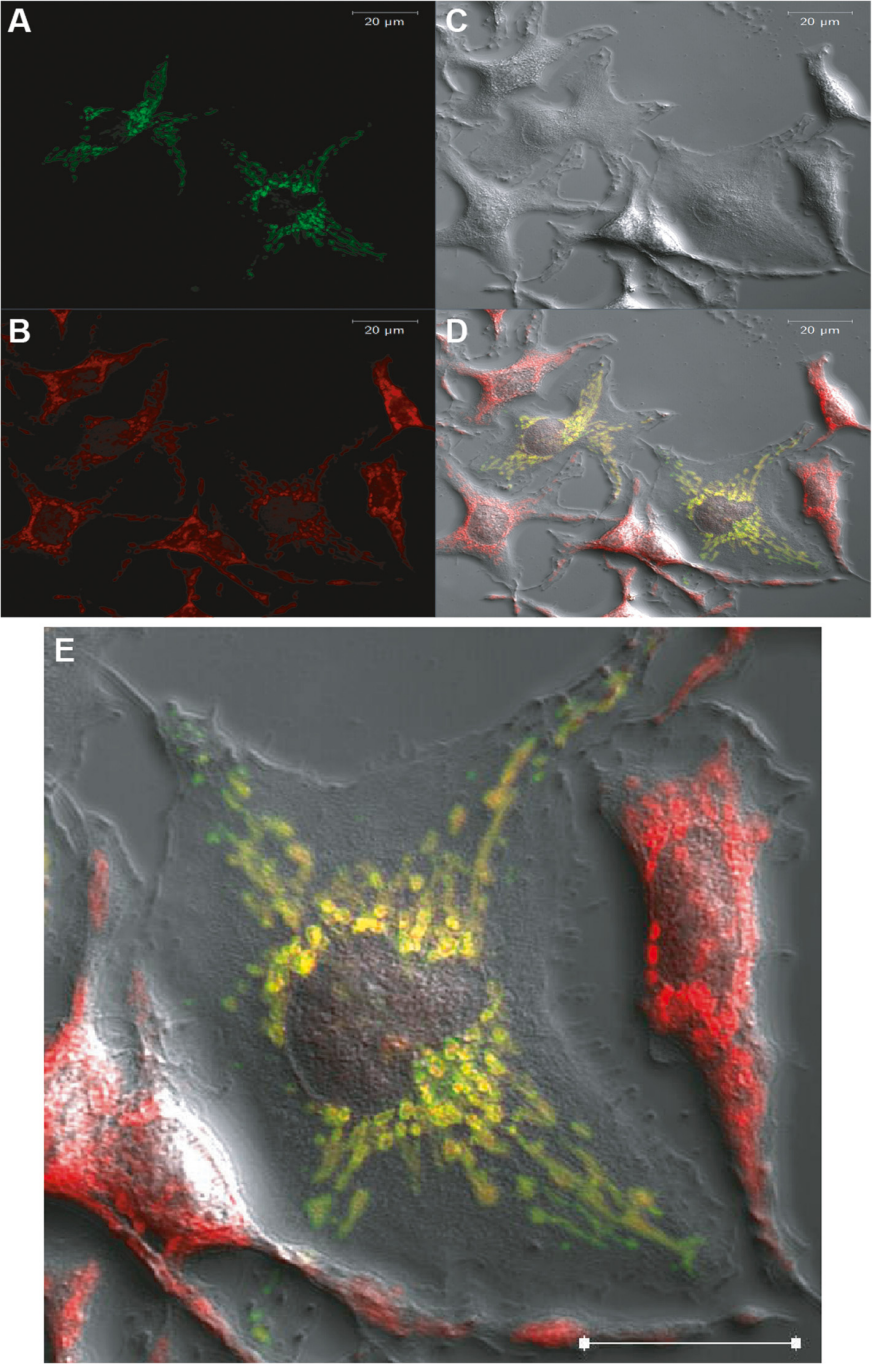
**Subcellular localization of TSPO-eGFP in RBL-2H3 cells.** RBL-2H3 cells were transiently transfected with TSPO-eGFP and stained with MitoTracker Red CMXRos. TSPO-eGFP **(A)** and MitoTracker **(B)** fluorescence were detected approximately 48 h after transfection. Resulting signals were analyzed and merged **(D)** with Zeiss ZEN 2009 software. Brightfield images **(C)** of the cells were added for reference. White bars (upper right corner) equal 20 μm. Similar results were obtained for at least 10 different cells from 3 independent experiments. **(E)** Image magnification of (**D**) showing one of the central cells to enable better visual analysis of the plasmalemnal area.

The likely interference of Ro5-4864 with the SFK Lyn does not entirely exclude a role for TSPO in MC activation. TSPO is mainly expressed in the OMM. However, in particular cell types TSPO expression has also been detected in the plasma membrane [21,22]. Since we observed fast and marked inhibitory effects of Ro5-4864 treatment on various signaling events and effector functions in MCs stimulated via different plasma membrane receptor systems, we sought to address the localization of TSPO in MCs by means of heterologous expression of a fluorescent TSPO fusion protein and confocal microscopy. Plasma membrane localization of TSPO could hint at direct interference with plasma membrane-resident receptors. A C-terminal eGFP fusion protein with TSPO was constructed, expressed in RBL-2H3 MCs or BMMCs and detected by confocal microscopy. Figure 8 displays in addition to a brightfield image of the RBL-2H3 cells (C) the signal from TSPO-eGFP (A), MitoTracker (B) and merge (D & E). The confocal images showed clearly that TSPO localizes to the mitochondria of RBL-2H3 cells. TSPO could not be detected in the plasma membrane. A TSPO-eGFP signal was absent in the plasma membrane even under the use of much higher laser intensities (data not shown). Compared to TSPO-eGFP, the eGFP control stained the cytoplasm evenly as expected (data not shown). The evaluation of TSPO-eGFP and mitochondria colocalization in BMMCs displayed similar results to RBL-2H3 cells. BMMCs are generally more difficult to examine under the microscope because they are substantially smaller than RBL-2H3 cells, are not adherent, and usually contain only little cytoplasm due to the considerable amount of space occupied by secretory granules and the nucleus. Nevertheless, colocalization of TSPO-eGFP and mitochondria was observed in BMMCs. As for RBL-2H3 cells, plasma membrane-resident TSPO could be excluded (Additional file 2: Figure S2). Interestingly, in all cells examined mitochondria appeared concentrated in a perinuclear region.

## Discussion

In the present study, our aim was to analyze molecularly the inhibitory effect of BDZs on MC activity by comparison of the effects of the two BDZs Ro5-4864 and clonazepam. The two drugs differ markedly in their affinities for the archetypical BDZ recognition sites, i.e., the GABA_A_ receptor and the TSPO, previously termed peripheral-type BDZ receptor. Ro5-4864 is an agonist at TSPO and has only low affinity to the GABA_A_ receptor [39], whereas clonazepam is a high-affinity GABA_A_ receptor agonist, but has only low affinity for TSPO [12]. Ro5-4864 concentration-dependently inhibited Ag-triggered degranulation in BMMCs and PMCs, whereas clonazepam was essentially ineffective in this respect. In accordance with this observation, Ro5-4864 suppressed Ca^2+^ mobilization, production of ROS and activation of the PI3K pathway (as measured by phosphorylation of Akt at Ser473), which are all important signaling events in the positive regulation of the secretory response [26,27]. These data suggest that Ro5-4864 and structurally related compounds might be applicable as versatile MC stabilizing drugs in MC-dependent diseases, e.g., hypersensitivity diseases, asthma, and systemic MCAD [18,40]. This was also shown by the inhibition of allergen-induced bronchoconstriction in rat precision-cut lung slices. In this context it is interesting to note that Ro5-4864 did not change the basal activation of the MCs indicating a selective action of BDZs at (pathologically) activated MCs.

The question arises for the target sites at which Ro5-4864 and other 1,4-benzodiazepines mediate their inhibitory effects on MCs. Since the selective GABA_A_ receptor agonist clonazepam did not mimic the effects of Ro5-4864, an action of Ro5-4864 at classical GABA_A_ receptors is rather unlikely. One potential candidate structure is TSPO, a transmembrane protein located in the OMM and enriched in OMM-IMM contact sites. It is a central component of a multimeric protein complex, comprising amongst others TSPO, VDAC1, ANT, and PRAX-1, and is associated with the mitochondrial permeability transition pore (mPTP) [6]. Therefore, functions of TSPO in regulating apoptotic processes have been discussed. Indeed, Ro5-4864 has been reported to induce apoptosis in some human and murine cancer cell lines and thymocytes, in particular by interfering with the mitochondrial membrane potential [41-45]. In these studies, cells were treated with Ro5-4864 for many hours to days, i.e. for a much longer time span compared to our experiments, which were performed within minutes to 3 h. The analysis of the effect of Ro5-4864 on BMMC survival showed only subtle apoptotic effects after 24 h (data not shown) excluding apoptotic effects within the significant shorter time windows of our MC activation experiments. Since we were able to reduce the pre-incubation time with Ro5-4864 to 1 min without loosing inhibitory efficiency, a mechanism via plasma membrane-located target sites instead of mitochondria-resident TSPO seems more likely. Interestingly, in certain cell types TSPO expression has also been detected in the plasma membrane [21,22]. By expressing a fluorescent TSPO fusion protein and using confocal microscopy, however, we were not able to detect the TSPO-containing fusion protein in the plasma membrane of MCs. Though we consider the technique used sufficiently sensitive, we did not have the material to detect endogenous TSPO and, thus, cannot totally exclude expression of the endogenous protein in the plasma membrane.

The concentration-dependent inhibition of Ag-triggered degranulation by Ro5-4864 could be due to suppression of mitochondrial Ca^2+^ uptake. Ag-triggered degranulation is dependent on influx of extracellular Ca^2+^ ions through SOC channels [46,47]. We found that Ro5-4864 suppressed SOC entry, whereas intracellular Ca^2+^ release from the ER appeared unaltered. Recently, Farsky and colleagues reported on a similar attenuation of fMLP-induced Ca^2+^ mobilization in neutrophils by Ro5-4864 [48]. Optimal SOC entry requires efficient emptying of the ER. To prevent immediate re-uptake of Ca^2+^ into the ER via the SERCA, mitochondria are able to take up Ca^2+^ at the moment of release from the ER by the uniporter channel. Ro5-4864 treatment might suppress this mitochondrial Ca^2+^ uptake mechanism. If so, passive release of Ca^2+^ from the ER by means of treatment with the SERCA inhibitor thapsigargin should be independent of such a mitochondrial buffering mechanism. Indeed, thapsigargin-induced SOC influx was not suppressed by Ro5-4864 treatment, which would be in agreement with this idea of an interaction of Ro5-4864 with mitochondrial Ca^2+^ uptake.

However, the effects of Ro5-4864 on Ag-triggered signaling in BMMCs deficient for the negative regulator SHIP1 point to another target site of Ro5-4864. Intriguingly, whereas Ro5-4864 concentration-dependently suppressed degranulation in SHIP1-deficient BMMCs, Ag-triggered activation of Akt was not altered in these cells. Moreover, there was only a slight reduction of extracellular Ca^2+^ influx (Additional file 3: Figure S3). It is known that SHIP1-deficient MCs are less sensitive to drugs inhibiting PI3K compared to wild-type MCs [49]. Since Akt phosphorylation and Ca^2+^ mobilization are PI3K-dependent [26,49] suppression of PI3K activation by Ro5-4864 was not as effective in SHIP1-deficient BMMCs. These data suggested that Ro5-4864 very likely did not interfere with the mitochondrial Ca^2+^ buffering mechanism (such an effect should be observable in SHIP1-deficient BMMCs as well) and that a target site located more upstream should be involved in Ro5-4864-mediated regulation of the secretory response.

1,4-Benzodiazepines have been reported to inhibit SFKs [35], which are known to play multiple important roles in MC activation, in particular via the FcεRI [50]. Amongst the first signaling events in MCs in response to FcεRI crosslinking are activation of the SFK Lyn, subsequent tyrosine phosphorylation of the FcεRI β-chain and γ-chain ITAMs by Lyn and activation of the tyrosine kinase Syk via interaction with the phosphorylated γ-chains and phosphorylation by Lyn [34]. Moreover, immediate activation of the SFK Fyn leads to the activation of the PI3K pathway [51]. Thus, pharmacological interference with SFK activation would have a negative impact on most FcεRI-mediated signaling pathways. Indeed, Ro5-4864 attenuated Ag-triggered tyrosine phosphorylation events in both wild-type and SHIP1-deficient MCs. Using a GST-SH2(Lyn) fusion protein to pull-down specific tyrosine-phosphorylated proteins, reduced phosphorylation of FcεRIβ and Syk was observed, indicating early interference of FcεRI signaling by Ro5-4864. Though both FcεRIβ and Syk are known targets of Lyn, involvement of other SFKs cannot be excluded, all the more since particularly Fyn seems involved in regulation of Ag-triggered degranulation and activation of the PI3K pathway [51]. In this respect, enhanced Ag-induced phosphorylation of Akt in Lyn-deficient BMMCs was markedly suppressed by Ro5-4864, clearly indicating Lyn-independent effects of this BDZ and suggesting a Fyn-dependent effect (Additional file 4: Figure S4).

Whereas degranulation is a fast response after Ag triggering of MCs occurring within a few minutes, production of pro-inflammatory cytokines takes place with slower kinetics. Important signaling pathways for cytokine production downstream of the FcεRI include the PI3K, p38, and NFκB pathways [33]. All of these pathways were attenuated by Ro5-4864 treatment in wild-type MCs underlining the role of central signaling elements, e.g. SFKs, being blocked by this drug. Intriguingly, Ro5-4864 also concentration-dependently suppressed cytokine production in response to stimulation of receptor systems such as Kit and TLR4. Also this effect can be explained by the inhibition of SFKs. Both Lyn and Fyn have been reported to play positive regulatory roles in the context of Kit signaling [52,53]. In addition, a recent publication by Avila et al. has demonstrated the importance of Lyn for the production of TNF-α in response to LPS in MCs [54]. Thus, all effects observed in the present study with the 1,4-benzodiazepine Ro5-4864 are explainable by attenuation of SFK activity.

## Conclusions

In conclusion, the present data demonstrate that the 1,4-benzodiazepine Ro5-4864 significantly suppresses pro-inflammatory MC responses downstream of differential ligand/receptor systems, most likely by attenuating SFK activity by direct inhibition of the respective SFK and/or indirectly by acting at a so far unknown upstream plasmalemnal recognition site. Hence, Ro5-4864 and structurally related compounds might be applicable as effective MC stabilizing drugs in different MC-dependent diseases, such as allergies, asthma, and systemic MCAD. It is however mandatory to identify and characterize the direct molecular target(s) to exclude unwanted side effects on other immune and non-immune cells. For certain MC-dependent diseases, however, topical administration as cream, eye drops or nasal spray could be options for first applications.

## Material and methods

### Chemicals

Ro5-4864, clonazepam, DNP-HSA (containing 30–40 mol DNP per mole albumin), monoclonal IgE anti-DNP (SPE-7), ovalbumin, thapsigargin, and EDTA were purchased from Sigma-Aldrich, Munich, Germany. Fluo-3 AM, Fura Red AM, pluronic F-127, H_2_DCFDA, MitoTracker Red CMXRos, and recombinant mouse SCF were obtained from (Invitrogen, Karlsruhe, Germany). Monoclonal mouse anti-P-Akt (S473), monoclonal rabbit anti-P-Erk1/2 (T202/Y204), polyclonal rabbit anti-P-IκBαS32, and polyclonal rabbit anti-P-p38 (T180/Y182) antibodies were purchased from Cell Signaling Technology, Frankfurt, Germany, polyclonal rabbit anti-p85 from Millipore, Schwalbach, Germany, polyclonal anti-Syk antibody (N-19) from Santa Cruz Biotechnology, and DMSO from AppliChem, Darmstadt, Germany. Monoclonal anti-FcεRIβ antibody was kindly provided by Dr. R. Siraganian (Bethesda, MD). R-form LPS from S. Minnesota mutant R595 was extracted and purified as described [55-57] and was a gift from Dr. M. Freudenberg and Dr. C. Galanos (MPI for Immunobiology and Epigenetics, Freiburg, Germany).

### Cell culture

Bone marrow-derived MCs (BMMCs): According to the technique established by Razin et al. [58], bone marrow cells (1×10^6^/ml) from 6 to 8 week old mice (129/Sv × C57BL/6) were cultured (37°C, 5% CO_2_) as single cell suspensions in RPMI 1640 medium supplemented with 15% FCS, 1% X63Ag8-653-conditioned medium (source of IL-3 [59]), 2 mM L-glutamine, 10 μM β-mercaptoethanol, 50 units/ml penicillin, and 50 mg/ml streptomycin. At weekly intervals, the non-adherent cells were reseeded at 5×10^5^ cells/ml in fresh medium. After 4–6 weeks in culture, greater than 99% of the cells were Kit and FcεRI positive as assessed by FACS using phycoerythrin-labeled anti-c-kit antibodies (Pharmingen, Mississauga, Canada) and FITC-labeled hamster anti-mouse FcεRIα antibodies (eBioscience, Frankfurt, Germany), respectively. SHIP1+/+ and −/− BMMCs as well as Lyn+/+ and Lyn−/− BMMCs were differentiated *in vitro* using the same protocol but starting from bone marrow cells of 6 to 8 week old SHIP1+/+ and −/− littermates (129/Sv × C57BL/6). Peritoneal MCs (PMCs) were cultivated according to Malbec et al. [23]. RBL-2H3 cells were maintained (37°C; 5% CO_2_) in RPMI 1640 medium supplemented with 10% FCS, 2 mM L-glutamine, 50 units/ml penicillin, 50 mg/ml streptomycin, and 50 μM β-mercaptoethanol.

### Cellular stimulation and western blotting

IgE-loaded BMMCs were washed in PBS and resuspended in RPMI/0.1% BSA. Cells were adapted to 37°C for 15 min and pretreated and stimulated as indicated. After stimulation, cells were pelleted and solubilized with 0.5% NP-40 and 0.5% sodium deoxycholate in 4°C phosphorylation solubilisation buffer [60]. After normalizing for protein content, the postnuclear supernatants (obtained after centrifugation at 4°C at 13,200 rpm in an Eppendorf 4515R centrifuge (F45-24-11 rotor) for 15 min) were subjected directly to SDS-PAGE and Western blot analysis [60]. The GST-SH2(Lyn) construct was described previously [61] and production, pull-down and immunoblotting were performed as published [62].

### Degranulation assay

For degranulation studies, MCs were preloaded with 0.15 μg/ml IgE anti-DNP overnight at 37°C. The cells were then washed and resuspended in Tyrode’s buffer (130 mM NaCl, 5 mM KCl, 1.4 mM CaCl_2_, 1 mM MgCl_2_, 5.6 mM glucose, and 0.1% bovine serum albumin (BSA) in 10 mM Hepes, pH 7.4). The cells were adapted to 37°C for 20 min and then treated at 37°C as mentioned. Vehicle (DMSO) and BDZ treatment was for 20 min prior to Ag (DNP-HSA) addition. The degree of degranulation was determined by measuring the release of β-hexosaminidase [63].

### Preparation and use of precision-cut lung slices

Precision-cut lung slices (PCLS) were prepared from 8-week-old Wistar rats (220 ± 20 g) obtained from Charles River (Sulzfeld, Germany) and kept under controlled conditions (22°C, 55% humidity and 12-h day/night rhythm). Animal experiments were approved by the local ethics committee. Rat PCLS were prepared as previously described [64]. Rats were sacrificed by an overdose of pentobarbital i.p. (60 mg/kg). Isolated lungs were filled with pre-warmed agarose solution (0.75%) via the trachea and subsequently chilled with ice. Then lobes were separated and cut into 5 to 10 mm thick tissue segments from which cores were made along the airways, and then cut into 250 ± 20 μm thick slices (Alabama Research and Development, Munford, AL). For studies with ovalbumin, the lung slices were incubated overnight with cell culture medium containing 1% serum from actively sensitized rats, as previously done [64]. After overnight culturing, the airways in PCLS were imaged and digitized using a digital video camera. A control picture was taken before addition of DMSO or Ro5-4864 (10 μM, 30 μM, and 100 μM) and after addition of ovalbumin (10 μg/ml) frames were recorded every 30 s for 15 min. The images were analyzed by the image analysis program Optimas 6.5 (Optimas, Bothell, WA).

### IL-6/TNF-α ELISAs

Mouse IL-6 and TNF-α ELISAs (BD Pharmingen, Heidelberg, Germany) were performed according to the manufacturer’s instructions. Absolute levels of cytokines in culture supernatants varied between experiments/BMMC cultures. Qualitative differences, however, were consistent throughout the study. Experiments were done in triplicates and performed at least three times.

### Measurement of Ca^2+^ mobilization

IgE-preloaded BMMCs were washed with RPMI 1640 medium, resuspended at 5 × 10^6^ cells/ml in RPMI 1640 containing 1% FCS, 1.3 μM Fluo-3 AM, 2.7 μM Fura Red AM, and 0.1% pluronic F-127, and incubated for 30 min at 37°C. Cells were then pelleted, resuspended in RPMI 1640 containing 1% FCS and analyzed in a FACSCalibur flow cytometer (BD Biosciences) after the indicated stimulation procedures. The FACS profiles were converted to line graph data using the FlowJo analysis software (Treestar, Ashland, OR, USA).

### Flow cytometric analysis of ROS production

IgE-sensitized BMMCs were washed with PBS, resuspended in RPMI 1640/1% FCS (5 × 10^6^ cells/ml), and stained with the free radical-sensitive dye H_2_DCFDA (final concentration: 10 μM) for 30 min at 37°C in the dark. Subsequently, stimulus (antigen) was added and flow cytometric analysis of cell samples was carried out using a FACSCalibur (Beckton Dickinson, San Jose, USA). Data were processed by FlowJo analysis software.

### Molecular cloning and transfection

To obtain a fusion construct comprising murine TSPO and an enhanced green fluorescent protein (eGFP), TSPO cDNA at its 3^′^-end was fused to eGFP sequence. Murine TSPO full-length cDNA (FANTOM clone I830130P14) was obtained from imaGenes GmbH (Berlin, Germany) and plasmid pEGFP-N1 from Clontech Laboratories Inc (Mountain View, USA). The coding sequence of TSPO was inserted in-frame using EcoRI and BamHI restriction sites resulting in pEGFP-N1-TSPO. The final plasmid was controlled by DNA-sequencing. RBL-2H3 cells as well as BMMCs were transiently transfected with pEGFP-N1-TSPO via electroporation with the Neon Transfection System (Life Technologies GmbH, Darmstadt, Germany) according to the manufacturer’s instructions.

### Fluorescence microscopy

RBL-2H3 cells were detached from the plate, reseeded on cover slips in a 12-well plate and incubated for another 24 h. BMMCs (24 h post transfection) were transferred to a 12-well plate containing cover slips pretreated with 0.1% poly-L-lysine in PBS and were also incubated for further 24 h. 48 h after transfection, mitochondria of RBL-2H3 cells and BMMCs were stained with MitoTracker Red CMXRos. Cells were incubated for 30 min at 37°C with 200 nM MitoTracker in stimulation medium. Cells were then washed twice with PBS containing 9 mM CaCl_2_ and 5 mM MgCl_2_ and fixed with methanol for 20 min in the dark at RT. Background fluorescence was then quenched for 5 min with 50 mM NH_4_Cl in PBS containing 9 mM CaCl_2_, 5 mM MgCl_2_, and 0.1% (v/v) Triton X 100 at RT. Finally, cells were washed in water and mounted on a glass slide with one drop of Immunomount. The prepared slides were analyzed with a Zeiss LSM 710 (Carl Zeiss AG, Jena, Germany) confocal laser scanning microscope. All images were taken with a 63x oil immersion objective. For the fluorophores, the following lasers, excitation wavelengths, and detected range of emission wavelengths were used: eGFP (Laser: Argon; Excitation Wavelength: 488 nm; detected wavelength range: 493–574 nm) and MitoTracker Red CMXRos (Laser: DPSS 561–10; Excitation Wavelength: 561 nm; Detected Wavelength Range: 568–691 nm). During all measurements, the pinhole was set to 1 AU. The obtained images were analyzed with the Zeiss ZEN 2009 software.

### Statistical analysis

P values were calculated by the paired two-tailed Student’s *t* test. P values of * < 0.05, ** < 0.005, and *** < 0.0005 were considered statistically significant. ns, non-significant.

## Competing interests

The authors declare that they have no competing interests.

## Authors’ contributions

OSY, TW, KMN, MK, and CM performed the experiments as well as prepared the figures and data analyses. GM, FK, and BH contributed to the conception and preparation of the manuscript and its critical revision. MH conceived of the study, designed experiments and jointly wrote the manuscript. All authors read and approved the final manuscript.

## Supplementary Material

Additional file 1: Figure S1 Suppression of LPS-induced IL-6 production in SHIP1-deficient BMMCs by Ro5-4864. SHIP1-deficient BMMCs were pretreated with DMSO, Ro5-4864 or clonazepam for 20 min and subsequently stimulated with 5 μg/ml LPS for 3 h or left unstimulated. Subsequently, IL-6 concentrations in the supernatants were determined by ELISA. Each bar is the mean of triplicates ± SEM. Comparable results were obtained with cells from different cultures. Marks of significance (“asterisks”) relate to the respective vehicle (DMSO) control. Click here for file

Additional file 2: Figure S2 Subcellular localization of TSPO-eGFP in BMMCs. BMMCs were transiently transfected with TSPO-eGFP and stained with MitoTracker Red CMXRos. TSPO-eGFP (A) and MitoTracker (B) fluorescence were detected approximately 48 h after transfection. Resulting signals were analysed and merged (D) with Zeiss ZEN 2009 software. A brightfield image (C) was added for reference. White bars (lower left corner) equal 20 μm. Similar results were obtained for different cells from independent experiments. Click here for file

Additional file 3: Figure S3 Effect of Ro5-4864 on antigen-triggered Ca^2+^ flux in SHIP1-deficient BMMCs. Intracellular Ca^2+^ was measured in SHIP1-deficient BMMCs by flow cytometry using the Ca^2+^-sensitive fluorescent dyes fluo-3 and fura red. Steady-state fluorescence was determined for 1 min before 1 mM EDTA (first arrow) was added for 1 min to chelate extracellular Ca^2+^. Ro5-4864 (100 μM) or DMSO were added (second arrow) and incubated for 2 min. Cells were then stimulated with 200 ng/ml Ag (third arrow) and the resulting Ca^2+^ response derived from intracellular store depletion was measured for 3 min. Finally, 2 mM CaCl_2_ was added (fourth arrow) to replenish extracellular Ca^2+^ stores and the resulting SOC influx was measured for 2 min. Comparable results were obtained with cells from different cell cultures. Click here for file

Additional file 4: Figure S4 Ro5-4864 treatment attenuates activation of the PI3K pathway in Lyn-deficient mast cells. IgE-loaded Lyn+/+ and Lyn−/− BMMCs were pretreated for 20 min with 100 μM Ro5-4864 or the respective amount of DMSO and stimulated with Ag (DNP-HSA, 20 ng/ml) for 5 min or left unstimulated. Subsequently, cellular lysates were analyzed by immunoblotting using antibodies against P-Akt (upper panel) and p85 (lower panel, loading control). Click here for file
