# Supplementary figures and images for: The 1,4-benzodiazepine Ro5-4864 (4-chlorodiazepam) suppresses multiple pro-inflammatory mast cell effector functions
Source: Cell Commun Signal. 2013 Feb 20;11:13. doi: 10.1186/1478-811X-11-13 (PMC3598916; doi:10.1186/1478-811X-11-13)

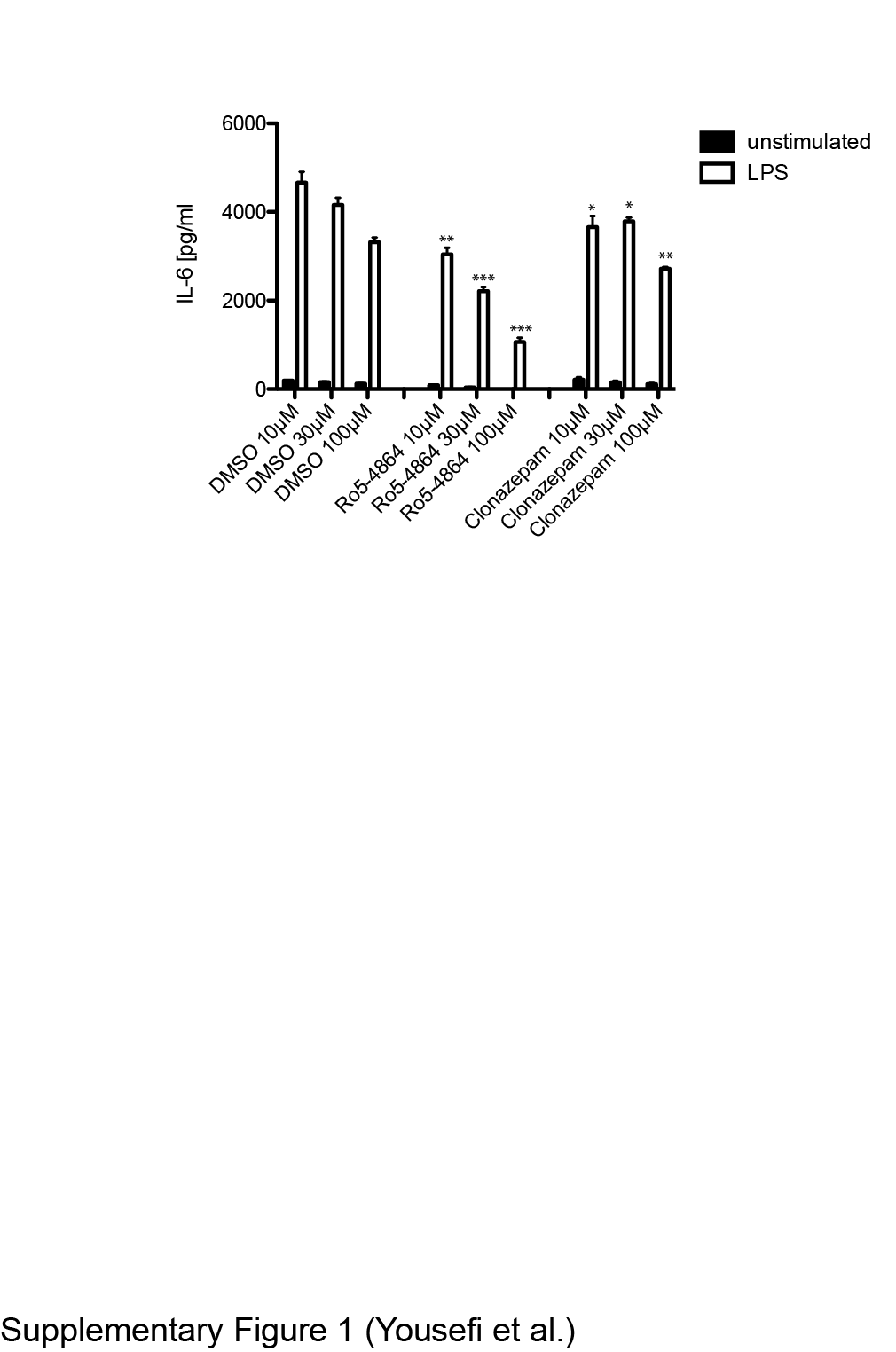

Supplement: Additional file 1: Figure S1 — Suppression of LPS-induced IL-6 production in SHIP1-deficient BMMCs by Ro5-4864. SHIP1-deficient BMMCs were pretreated with DMSO, Ro5-4864 or clonazepam for 20 min and subsequently stimulated with 5 μg/ml LPS for 3 h or left unstimulated. Subsequently, IL-6 concentrations in the supernatants were determined by ELISA. Each bar is the mean of triplicates ± SEM. Comparable results were obtained with cells from different cultures. Marks of significance (“asterisks”) relate to the respective vehicle (DMSO) control. [file 1478-811X-11-13-S1.tiff]

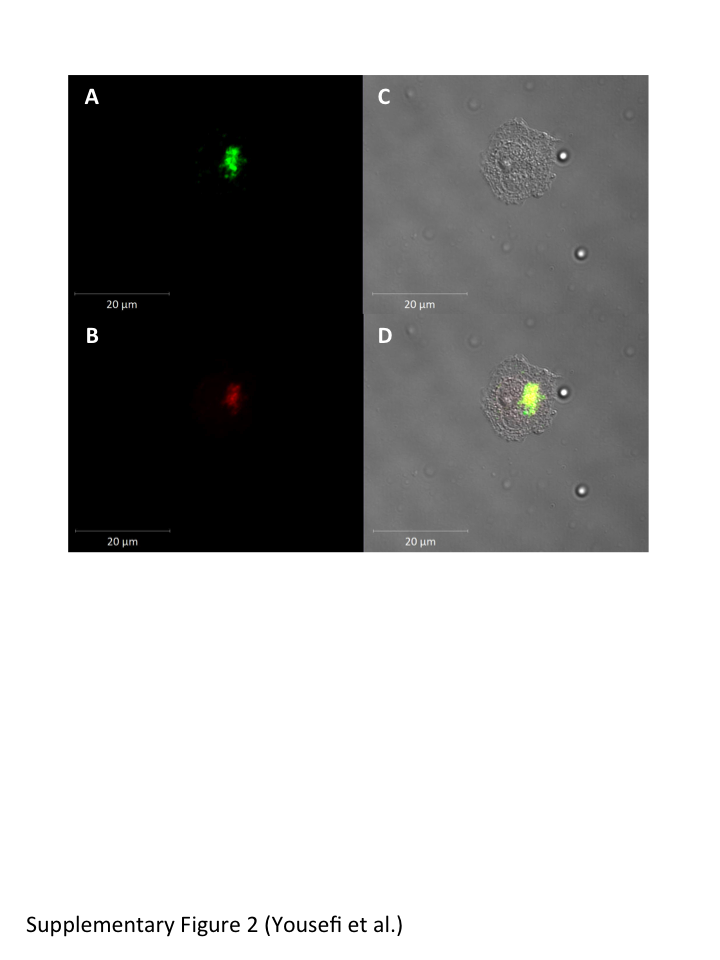

Supplement: Additional file 2: Figure S2 — Subcellular localization of TSPO-eGFP in BMMCs. BMMCs were transiently transfected with TSPO-eGFP and stained with MitoTracker Red CMXRos. TSPO-eGFP (A) and MitoTracker (B) fluorescence were detected approximately 48 h after transfection. Resulting signals were analysed and merged (D) with Zeiss ZEN 2009 software. A brightfield image (C) was added for reference. White bars (lower left corner) equal 20 μm. Similar results were obtained for different cells from independent experiments. [file 1478-811X-11-13-S2.tiff]

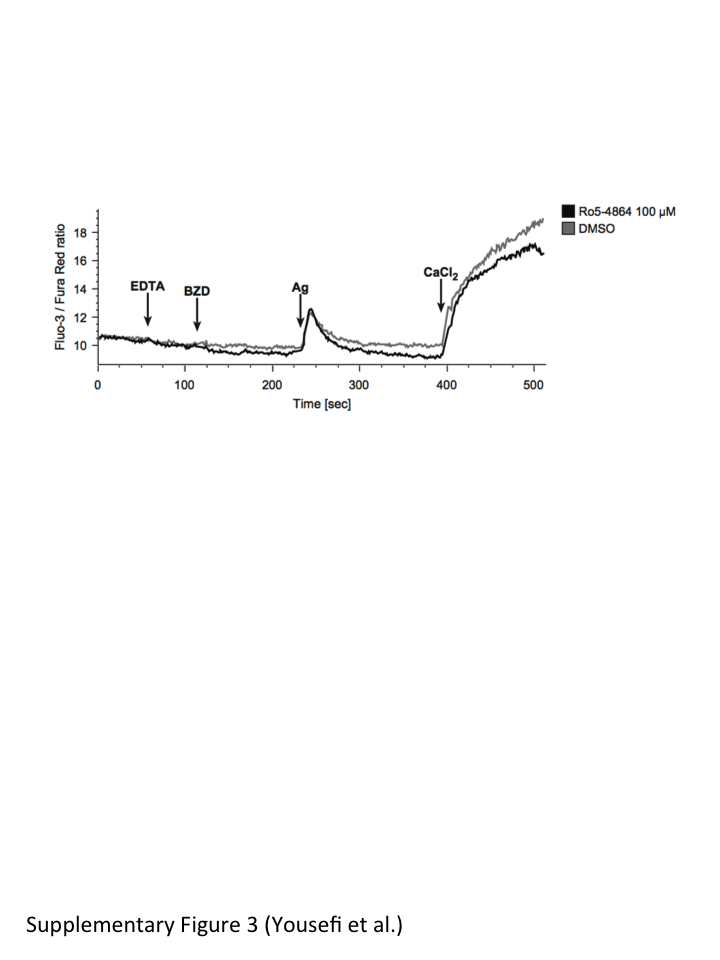

Supplement: Additional file 3: Figure S3 — Effect of Ro5-4864 on antigen-triggered Ca2+ flux in SHIP1-deficient BMMCs. Intracellular Ca2+ was measured in SHIP1-deficient BMMCs by flow cytometry using the Ca2+-sensitive fluorescent dyes fluo-3 and fura red. Steady-state fluorescence was determined for 1 min before 1 mM EDTA (first arrow) was added for 1 min to chelate extracellular Ca2+. Ro5-4864 (100 μM) or DMSO were added (second arrow) and incubated for 2 min. Cells were then stimulated with 200 ng/ml Ag (third arrow) and the resulting Ca2+ response derived from intracellular store depletion was measured for 3 min. Finally, 2 mM CaCl2 was added (fourth arrow) to replenish extracellular Ca2+ stores and the resulting SOC influx was measured for 2 min. Comparable results were obtained with cells from different cell cultures. [file 1478-811X-11-13-S3.tiff]

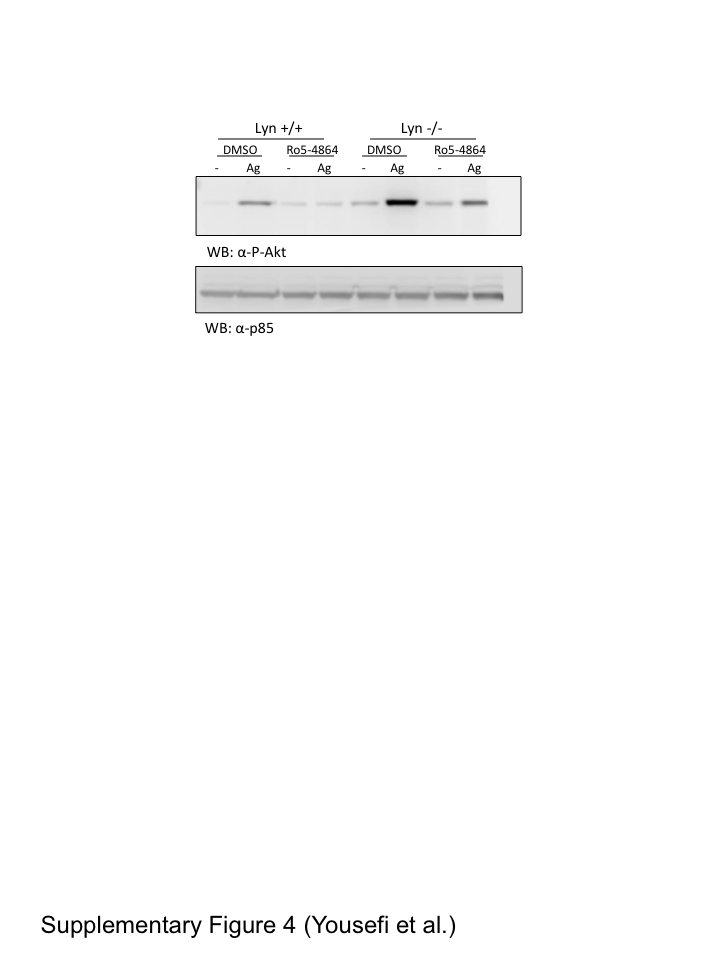

Supplement: Additional file 4: Figure S4 — Ro5-4864 treatment attenuates activation of the PI3K pathway in Lyn-deficient mast cells. IgE-loaded Lyn+/+ and Lyn−/− BMMCs were pretreated for 20 min with 100 μM Ro5-4864 or the respective amount of DMSO and stimulated with Ag (DNP-HSA, 20 ng/ml) for 5 min or left unstimulated. Subsequently, cellular lysates were analyzed by immunoblotting using antibodies against P-Akt (upper panel) and p85 (lower panel, loading control). [file 1478-811X-11-13-S4.tiff]
